# Locust Collective Motion and Its Modeling

**DOI:** 10.1371/journal.pcbi.1004522

**Published:** 2015-12-10

**Authors:** Gil Ariel, Amir Ayali

**Affiliations:** 1 Department of Mathematics, Bar Ilan University, Ramat-Gan, Israel; 2 Department of Zoology, Faculty of Life Sciences, and Sagol School of Neuroscience, Tel Aviv University, Tel Aviv, Israel; University of Utah, UNITED STATES

## Abstract

Over the past decade, technological advances in experimental and animal tracking techniques have motivated a renewed theoretical interest in animal collective motion and, in particular, locust swarming. This review offers a comprehensive biological background followed by comparative analysis of recent models of locust collective motion, in particular locust marching, their settings, and underlying assumptions. We describe a wide range of recent modeling and simulation approaches, from discrete agent-based models of self-propelled particles to continuous models of integro-differential equations, aimed at describing and analyzing the fascinating phenomenon of locust collective motion. These modeling efforts have a dual role: The first views locusts as a quintessential example of animal collective motion. As such, they aim at abstraction and coarse-graining, often utilizing the tools of statistical physics. The second, which originates from a more biological perspective, views locust swarming as a scientific problem of its own exceptional merit. The main goal should, thus, be the analysis and prediction of natural swarm dynamics. We discuss the properties of swarm dynamics using the tools of statistical physics, as well as the implications for laboratory experiments and natural swarms. Finally, we stress the importance of a combined-interdisciplinary, biological-theoretical effort in successfully confronting the challenges that locusts pose at both the theoretical and practical levels.

## Introduction—Why Locusts?

“The locusts have no king, yet go they forth all of them by bands” (Proverbs 30:27, *The Holy Bible*, *King James Version*. Cambridge Edition. 1611). The Old Testament, like other ancient scripts, has several references to locusts and their behavior, specifically to the spectacular phenomenon of marching locust hopper bands. Part of our fascination with locust swarms is due to the fact that they always have been and still are a major threat to agriculture (e.g., [1,2]). Locusts are described as a pest of unusually destructive powers: A desert locust adult can consume roughly its own weight, i.e., about two grams, in fresh food per day [3]. The notorious 1915 locust attack in the Middle East, for example, resulted in wiping out a largely underestimated 536,000 tons of food [4]. According to the Food and Agriculture Organization of the United Nations, in modern days, e.g., during the 2003–2004 locust invasions, this translated to around US$30 million spent by a typical African nation in anti-locust campaigns [5].

The coordinated activity of crowds consisting of millions of individuals, while not unique to locusts, has been and still is a challenge to laymen and scientists alike. Despite considerable progress in understanding the mechanisms underlying the emergence and synchronization among moving crowds of animals as well as humans, locust swarming still presents several fundamental open questions. These include key questions regarding locust biology as well as more theoretical aspects. Some of the open, or far from fully answered, questions include:

- What are the principle interactions between conspecifics in a swarm?
- What is the effect of the environment on the swarm and vice versa?
- What are the functional and evolutionary advantages to swarming?
- How do local dynamics within a swarm translate to macroscopic dynamics of large swarms consisting of millions of individuals?
- Do order and disorder in locust swarms constitute a phase transition in the sense of statistical physics, or are there metastable states of the dynamics?
- Are there quantifiable traits unique to locust swarms in respect to other animal crowds (fish, birds, humans, etc.)?

In the hope of encouraging more research into the above, as well as to provide a showcase of current knowledge, in this review, we summarize the different attempts to describe or capture locust collective behavior by way of theoretical modeling. As presented below, some of these efforts are heavily rooted in biological data and observations, while others offer a more mathematical or physical perspective. Some are specifically tailored and aimed toward explaining locust behavior; others are more general descriptions of collective motion, adapted to the locust case. The emphasis of the review is on marching locust nymphs. From the biological point of view, this is the critical stage during which the phenomenon of locust aggregation begins. Accordingly, recent years have seen several theoretical and modeling attempts to explain the emergence and maintenance of order in marching locust nymphs.

We start with the biological background: locusts, density-dependent phases in locusts, swarming, and collective movement (marching and flying). We then move on to present some of the attempts to model locust collective behavior, from the early models of Self-Propelled Particles (SPPs) to our own recent attempt based on intermittent pause-and-go motion. Models of flying swarms, locust phase change, and evolutionary models are also briefly surveyed. We conclude with a discussion of the success (or failure) of theoretical models in addressing the key questions presented above, as well as the benefits of such models in dealing with the locust problem.

## Biological Background

### Locust and locust density-dependent phase polyphenism

Locusts are short-horned grasshoppers that exhibit density-dependent phase-polymorphism or polyphenism (the phenomenon in which two or more distinct phenotypes are produced by the same genotype). They appear in two forms or phases [6]: crowding induces the gregarious phase, most notorious for its tendency to aggregate and form massive swarms; while isolation leads to the solitary phase, in which individuals actively avoid other locusts. The differences between the gregarious and solitary phases are collectively termed phase characteristics, extending from behavior and ecology, through morphology and anatomy, to physiology and biochemistry (see recent reviews in [7,8]). Locust phase polyphenism is continuous, and many intermediate forms can be found between the extreme gregarious and solitary phases. The phase transformation, i.e., the response to changes in population density, is reversible and not developmental-stage- or age-specific. Some phase characteristics, especially behavioral patterns, respond rapidly within the same instar (larval stage). Other characteristics, for example, color changes, only show an overt response in the next or subsequent instars. Parental density affects the color of the hatchlings and may have some behavioral and further effects on the next generation ([9–12], and see [13] for a review). Full-scale phase transformation takes several generations, and presumably occurs only in the field. In the laboratory, locusts kept either under crowding or under isolation, respectively approach the characteristics of the gregarious and the solitary phases [8].

Some 15 locust species, belonging to several, sometimes distant, phylogenetic groups, are considered true locusts, i.e., they express density-dependent polyphenism, swarming, and migration (see [6,8,14] and references within). Most notorious (and, thus, most studied) is the desert locust (*Schistocerca gregaria*), which inhabits dry grasslands and deserts and has threatened agricultural production in Africa, the Middle East, and Asia for centuries. Hereon, unless explicitly noted, we will mostly refer to the desert locust, as this is the most investigated species and a major part of our knowledge is based on it. The range of the migratory locust (*Locusta migratoria*) is wider than that of any other. It is found in grasslands throughout Africa, major parts of Eurasia, the East Indies, tropical Australia, and New Zealand. The Australian plague locust (*Chortoicetes terminifera*) is the most important pest species of locust in Australia, due to the large areas infested, the frequency of outbreaks, and its ability to produce several generations in a year. These, as well as other, less prominent locust species, may differ in their ability to express the phase phenomenon in its entirety, in the dynamics and magnitude of their response to changes in population density [8,14,15]. This important point has been largely overlooked by biologists and even more by theoreticians, tempted to generalize from a single-species study to all locusts. As our knowledge of diverse species increases, we may be introduced to key species-specific differences in the dynamics of the phase phenomenon [6,8–10]. The intricate phylogeny of locusts also offers a challenge to any attempt at explaining the evolution of the locust phenomenon (see the section “Evolution of the Locust Phenomenon” below).

**Fig 1:**
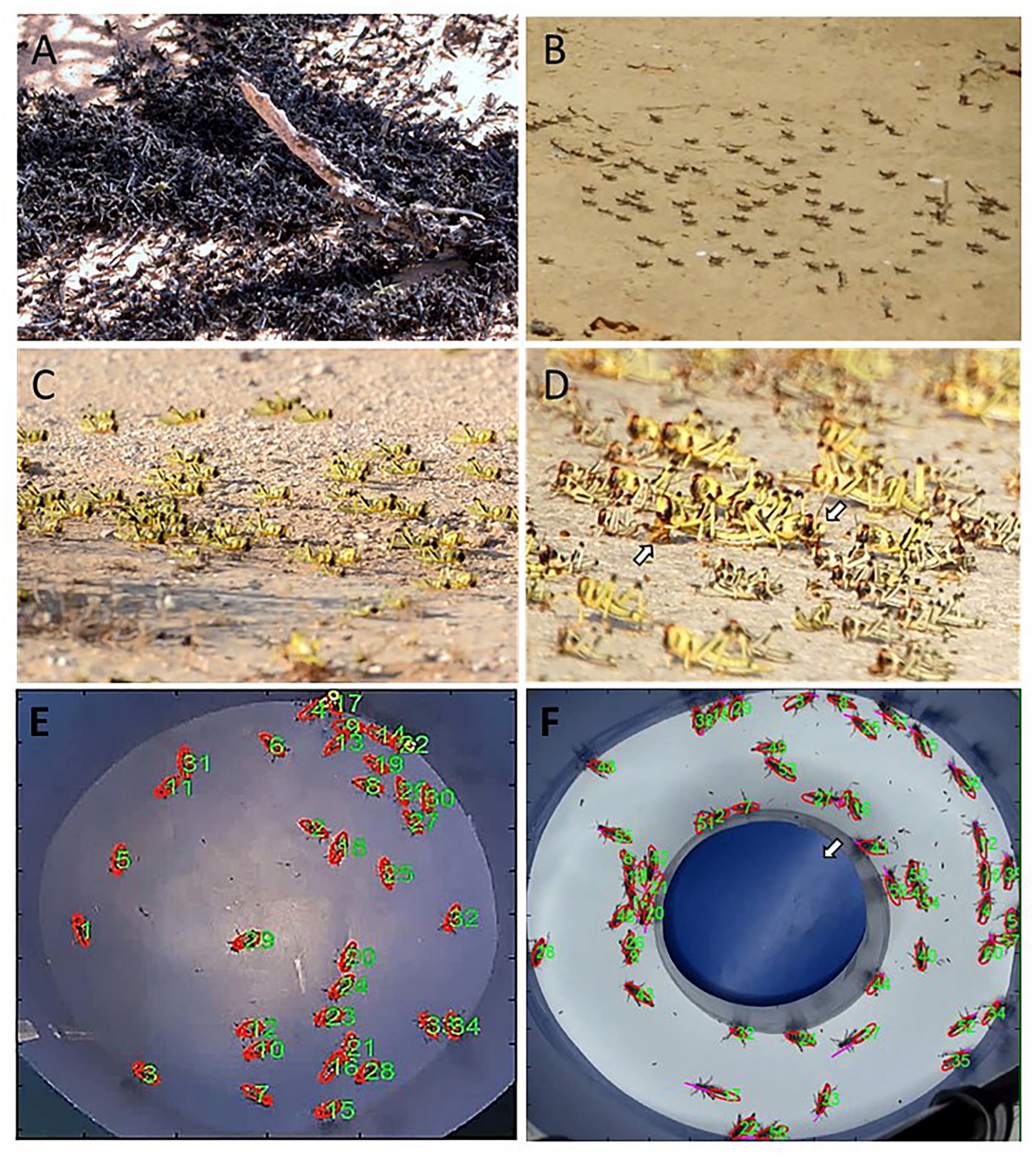
(A) A major behavioral characteristic of locusts in the gregarious phase is their strong propensity to form mass aggregations, as demonstrated by these desert locust nymphs. (B) Both swarming and marching start very early, already a few days after hatching, as demonstrated by these first larval instar desert locusts. (C) The endless marching bands of locust nymphs exemplify extreme coordination in their movement vectors during collective motion. (D) Road kills (nymphs hit by passing cars while the swarm crosses a road) will be immediately cannibalized by others (arrows). Locusts stopped in the midst of the swarm to feed on the cadavers will be totally ignored by others. Continuous and careful monitoring of individuals within a crowd in controlled lab experiments (E and F) have enhanced the development of quantitative analysis and theoretical modeling of individual dynamics and interactions, leading to various models of coordinated collective behavior.

As mentioned, locust behavior is a prominent phase characteristic. The importance of the change in behavior is that it precedes and facilitates all other phase changes. The major behavioral characteristic of locusts in the gregarious phase is their strong attraction to their conspecifics, which translates to active aggregation behavior (Fig 1A) [6,16,17]. Gregarious locusts are also generally more active, including their strong propensity to march in huge bands of hoppers or to form flying swarms as adults. In contrast, solitary-reared locusts actively avoid contact with other locusts [18–20]. They are more sedentary and cryptic in behavior, do not march, and fly less. The behavioral phase transformation is a positive-feedback process. Solitary-reared locusts have been reported to acquire most of the behavioral characteristics of the gregarious phase within 4–8 hours of crowding, including the propensity to aggregate, and thus reinforce the gregarizing stimuli provided by other locusts [19]. Recently, it has been shown that even a 30-minute exposure of a solitary-reared nymph to a small crowd of locusts is sufficient for the induced change in behavior to persist, even after re-isolation of the locust for 24–72 hours [20]. Details of the dynamics of phase transformation and its interactions with the environment are still far from being fully resolved [8].

### Locust swarming and coordinated movement

As mentioned, swarming is a hallmark of locust behavior. Swarming per se is a result of the attraction and aggregation tendency of locusts in the gregarious phase. Locust-coordinated mass movement includes the flight of adult swarms, but even more conspicuous is the marching of nymphs or hopper bands. It should be noted, however, that the formation of dense bands of hoppers is independent of the marching behavior: the multitude of different behavioral patterns of the bands include other, relatively quiescent, states, such as basking, perching, sheltering, and, of course, feeding. Hopper densities in quiescent bands are extremely high (Fig 1A), and the area that a quiescent band covers can be 2–4-fold smaller than that covered by the same band when marching, as reported for desert locusts by Ashall and Ellis [21,22], with even greater differences reported for other locusts ([23] and references therein).

Both swarming and marching start very early on, at the first larval instar, a few days after hatching (Fig 1B). Desert locust nymphs (as well as other locusts) have a diurnal pattern of behavior (e.g., [24,25]). The locust bands will normally have one period of marching in the forenoon and another in the afternoon, strongly dependent on weather conditions such as temperature, but also on clouds obscuring the sun, as well as on rain or cold wind [23]. In our own observations of the desert locust (Fig 1; Negev desert, Israel, spring of 2013; Ayali unpublished), marching usually took place from just before noon to two hours before sunset. The marching hopper bands can cover very different areas, from a few hundred square meters up to several kilometers. As reported for different locust species, their shape can also vary, ranging from columnar to frontal structures [23,25]. During marching of the hoppers, bands may meet, merge, or fuse, and the coordinated movement will thereby further enhance the swarming (this is also true for the flying swarms).

Undisturbed nymphs within marching bands will advance predominantly by very consistent walking rather than by jumping or hopping (the latter are aversive reactions, most commonly demonstrated in response to danger or threat [26–29]). This behavior is most typical to the desert and migratory locusts and may be less so in other species. Regarding the speed of marching, Uvarov [23] had already noted that “the actual speed of individual hoppers bears only a remote and uncertain relation (by a factor of 3–4) to the marching rate of the bands.” The reason for this is that the proportion of hoppers marching at any given moment can be as low as 10%. This has been confirmed by others (cited therein, and see also [24]) and by our own observations: individual marching locusts very often stop for periods of various durations. Of special interest is the behavior of hoppers at the edges of the marching band. Again, according to Uvarov ([23], and references within), hoppers advancing beyond the edge of a marching band frequently stop or even turn back to rejoin it. Similar observations were reported by Ellis and Ashall [24]. In their detailed description of marching bands of the Australian plague locust, Buhl et al. [30] refer specifically to the front edge of the band, finding that locusts at the front will slow down or simply turn more often, reducing their net displacement in comparison to those behind (as also reported for fish schools [31,32]). These behaviors will serve to maintain the integrity of the marching bands. They often result in bands comprising an extremely dense front followed by an exponential decay of density, with a consequent loss of cohesion toward the back [30]. What the major factors are that affect the direction of marching is still an open question.

The cannibalistic propensity of locusts has been recently associated with marching behaviour and was even suggested as a major driving force for the formation of collective motion in locust nymphs [33–35]. The controlled experiments and intriguing results of Bazazi et al. (e.g., [33]) indicate reduced marching of nymphs in an experimental arena following manipulation of sensory inputs from behind (visual and/or tactile). These reports are a good example of experimental results directly motivating a modeling approach, as discussed in the section “Escape and pursuit” below. They also underlie a putative theory for the evolution of phase polyphenism (see “Evolution of the Locust Phenomenon”). Cannibalism is very well documented in locusts (and other grasshoppers), e.g., [22,36–38]. However, it is accepted by most authors that cannibalism is mainly directed toward hatchlings [22,39], freshly molted [40], injured, or dead animals [37], and is rarely directed toward active, healthy individuals. Moreover, the documented dynamics within the marching band as described above (pauses of varied length, behavior at the band edges, etc.) are not consistent with the nymphs escaping predation or trying to cannibalize on their marching companions. The results of Bazazi et al. [33] may also be interpreted as merely supporting an instrumental role of visual inputs in the local interactions among locusts (see also 3.2 below), as well as confirming the cannibalistic tendency toward unhealthy individuals (surgically manipulated in this case).

While Uvarov [3] goes as far as to categorically reject any connection between ground movement and hunger (page 83 therein), it is well established that the nutritional state of the hoppers does have an important impact on the amount of marching, its speed, and the distance travelled [34,41]. Hence, it may be suggested that in addition to possible species-specific differences, different interactions among locust nymphs may occur at different behavioral contexts, and maybe also at different times of the day (e.g., some effect of a cannibalistic drive during the onset of marching in the morning, but non-cannibalistic interactions when marching is established or during high-density aggregation in vegetation during the hottest period of the day, etc.).

Flying swarms of adult locusts will migrate predominantly downwind. There are very few studies focusing on local dynamics within the flying swarm. Within a given section of the swarm, locusts were observed to generally fly with their bodies parallel, separated by as little as 10 cm [42–45]. Camhi et al. [46] even reported wing-beat coupling among pairs of locusts flying in tandem. Although aligned with their neighbors, locust groups within the body of the swarm head in random directions [43]. The integrity of the swarm is maintained by the fact that, similar to the marching hoppers, upon reaching the edges of the swarm locusts will turn back to re-join it. Hence, in the flying swarms, it is not clear whether and how local interactions and dynamics translate to coordinated swarm locomotion.

### Experimental approaches to the study of locust swarming

Traditionally, studies of locust behavior have been limited to field observations during locust outbreaks in the affected African regions. Such reports include the pioneering work of Uvarov [6,23,47], Kennedy [48,49], and others. These efforts have been aided by the development of techniques that enabled the investigation of particular aspects of the behavior of individual locust hoppers in bands (e.g., the photographic techniques of Stower [50]). A crucial step towards a more advanced understanding of coordinated behavioral mechanism in locusts was made by the first laboratory experiments, including the generation of marching behavior in closed circular arenas and the experimental manipulation of locust density [16,17,51–53]. These early reports already identified the strong attraction of gregarious locusts to conspecifics, which translates into active aggregation behavior; and, furthermore, already suggested that locust collective movement is highly dependent on the density of animals in the group.

Behavioral experiments were supplemented by extensive literature on locust physiology and neurobiology (e.g., [54], and references within). Most significant were advances in describing neurobiological correlates to locust phase polyphenism (starting with [55,56]), as well as attempts at identifying the neurobiological-related mechanisms of the phase change (reviewed in [57]). These have disclosed relevant sensory modalities that may be important in the complex interactions between individuals within a locust swarm.

Using modern computer-vision tracking methods (Fig 1E and 1F), locust marching behavior in the controlled environment of the lab has lent itself easily to continuous and careful monitoring of the crowd and the individuals, and to quantitative analysis. In the experiments by Buhl et al. [58], groups of up to 120 insects were placed in a ring-shaped arena with an external diameter of 80 cm. Locusts were allowed to march freely for several hours, monitored by a video camera. The films were later analyzed to produce individual trajectories, allowing a detailed quantitative analysis of the swarm dynamics. Recently, Ariel et al. [59] used similar experiments to investigate the animal–animal interaction in order to reveal some of the microscopic details of locust motion within a crowd—when does it walk, pause, or turn? A surge in such quantitative studies of locust coordinated behavior in the past decade or two (see below) have kept locust studies up-to-date and relevant and ensured their place at the forefront of the interdisciplinary efforts to unravel the secrets behind collective animal behavior.

## Modeling Marching Locust Nymphs

The past years have witnessed ample theoretical work on collective motion. A large number of recent reviews describe a wide range of theoretical approaches, suggesting explanations for the emergence of collective motion in animals (e.g., [60–67]). In general, modeling approaches can be categorized as either continuous models, written in terms of integro-differential equations, or discrete agent-based models.

Continuous models typically describe the coarse-grained density of animals and other system constituents as continuous fields, for example, by coupled reaction-diffusion equations or, following a kinetic approach, by hydrodynamic or Boltzmann equations [60–68]. One of the main drawbacks of continuous models is the difficultly of relating actual properties of individual animals (e.g., body shape and size, hunger, and other internal states) to specific details of the model [60]. This is one of the reasons why most of the current theoretical work on locust collective motion comprises agent-based simulations, which are useful for describing the dynamics from the point of view of the individual animal (“Umwelt” in biology [69], or “Lagrangian description” in physics [60]). The dynamics in agent-based models, also known as SPPs, are given by specifying the internal state of each animal, its interaction with others (conspecifics), and its interactions with the environment. However, such models are limited by the number of agents that can be simulated (very far, for example, from the millions of individuals comprising a locust swarm). In addition, they do not provide a macroscopic, or coarse-grained, description of the swarm dynamics as a whole, and additional mathematical tools may be needed to interpret the results [70,71].

Attempts to capture the individual dynamics leading to locust swarming behavior can be categorized into four major directions. The first is largely based on or derived from the work of Czirók et al. [72]; the second is our own recent attempt, emphasizing an intermittent pause-and-go motion pattern locust exhibit. In the following, we describe a general framework that allows a quantitative comparison between the different models in these two categories, their predictions as to order-disorder transitions, as well as their success in describing experiments. The third approach is derived from the widely-used three-zone, or Avoidance–Alignment–Attraction (AAA), model. The fourth approach, referred to as “escape and pursuit” (E&P) [73], is based on a theory of cannibalism being a dominant trait in locust behavior. We critically and comparatively describe these modeling approaches below.

### Locusts as self-propelled particles

The experiments by Buhl et al. [58] of locust marching in a circular arena motivated increased theoretical interest in the field. One of the main conclusions of this work was that the “swarm” synchronizes in either clockwise (CW) or counter-clockwise (CCW) movement. Furthermore, the time spent in an ordered state (a synchronized direction) increased with the number of animals, as switching from CW to CCW and vice-versa become rare events. The ring-shaped arena and directional switching motivated Buhl et al. to suggest a one-dimensional (1D) model (referred to hereafter as the Buhl model) adapted from Czirók et al. ([72]; referred to hereafter as the Czirók model). The applicability of a relatively simple 1D model to a real-life experiment quickly attracted interest by the theoretical community.

The Czirók model is essentially a 1D version of the well-known 2D Scalar Noise Model (SNM) of Viscek et al. [74]. We begin here with a description of the model that is continuous in time. Consider *N* particles with positions *x*(*t*) = (*x*_1_(*t*),…,*x*_*N*_(*t*)) and dimensionless velocities *u*(*t*) = (*u*_1_(*t*),…,*u*_*N*_(*t*)) moving along a line of length *L* with periodic boundaries. In order to facilitate comparison between the model and its many variants, we write the dynamics as a system of Stochastic Differential Equations (SDEs),

$$\begin{array}{l} {\dot{x}}_{i}=vu_{i}\\ {\dot{u}}_{i}=\left[G\left({\langle u(t)\rangle }_{i}\right)-u_{i}\right]+\sigma {\dot{W}}_{i}, \end{array}$$ (1)

where, ${\dot{W}}_{i}$ is white noise (independent for each *i*), *v* parameterizes the speed of particles, and *σ* is the standard deviation of external noise. The interaction of a focal particle *i* with its conspecifics is assumed to depend only on the local average direction within an interaction distance Δ,

$${\langle u(t)\rangle }_{i}=\{\begin{array}{cc} \frac{1}{n_{i}(t)}\sum_{j\in A_{i}(t)}u_{j}(t) & n_{i}(t)\geq 1\\ 0 & n_{i}(t)=0, \end{array}$$

where *A*_*i*_(*t*) denotes the set of neighbors of particle *i* at time *t* up to an interaction distance Δ, *A*_*i*_(*t*) = {*j*:|*x*_*i*_(*t*)-*x*_*j*_(*t*)|≤Δ} and *n*_*i*_(*t*) = |*A*_*i*_(*t*)| is the number of neighbors. In the Czirók model, as in most later variations of this model, the function *G*(·) is taken to be an odd, piece-wise continuous function,

$$G(u)=\{\begin{array}{cc} \frac{1}{2}\left[u+\text{sign}(u)\right] & u\neq 0\\ 0 & u=0. \end{array}$$

Discretizing (Eq 1) using Euler-Maruyama with step-size Δ*t*,

$$\begin{array}{l} x_{i}(t+\Delta t)=x_{i}(t)+vu_{i}(t)\Delta t\\ u_{i}(t+\Delta t)=u_{i}(t)+\left[G\left({\langle u(t)\rangle }_{i}\right)-u_{i}(t)\right]\Delta t+\sqrt{\Delta t}\xi_{i}(t), \end{array}$$ (2)

where *ξ*_*i*_(*t*) are independent random variables with zero mean and variance *σ* ^2^. Taking uniformly distributed noise, *ξ*_*i*_(*t*)~*U*[-*η*/2,*η*/2], $\eta =\sigma^{2}/\sqrt{12}$ yields the model described by Yates et al. [75]. Taking Δ*t* = 1 yields the discrete Czirók model,

$$\begin{array}{l} x_{i}(t+1)=x_{i}(t)+vu_{i}(t)\\ u_{i}(t+1)=G\left({\langle u(t)\rangle }_{i}\right)+\xi_{i}(t). \end{array}$$

In order to analyze and quantify order in the system, consider an order parameter *ϕ*(*t*) defined as

$$\phi (t)=\frac{1}{N}\sum_{i}u_{i}(t).$$

**Fig 2:**
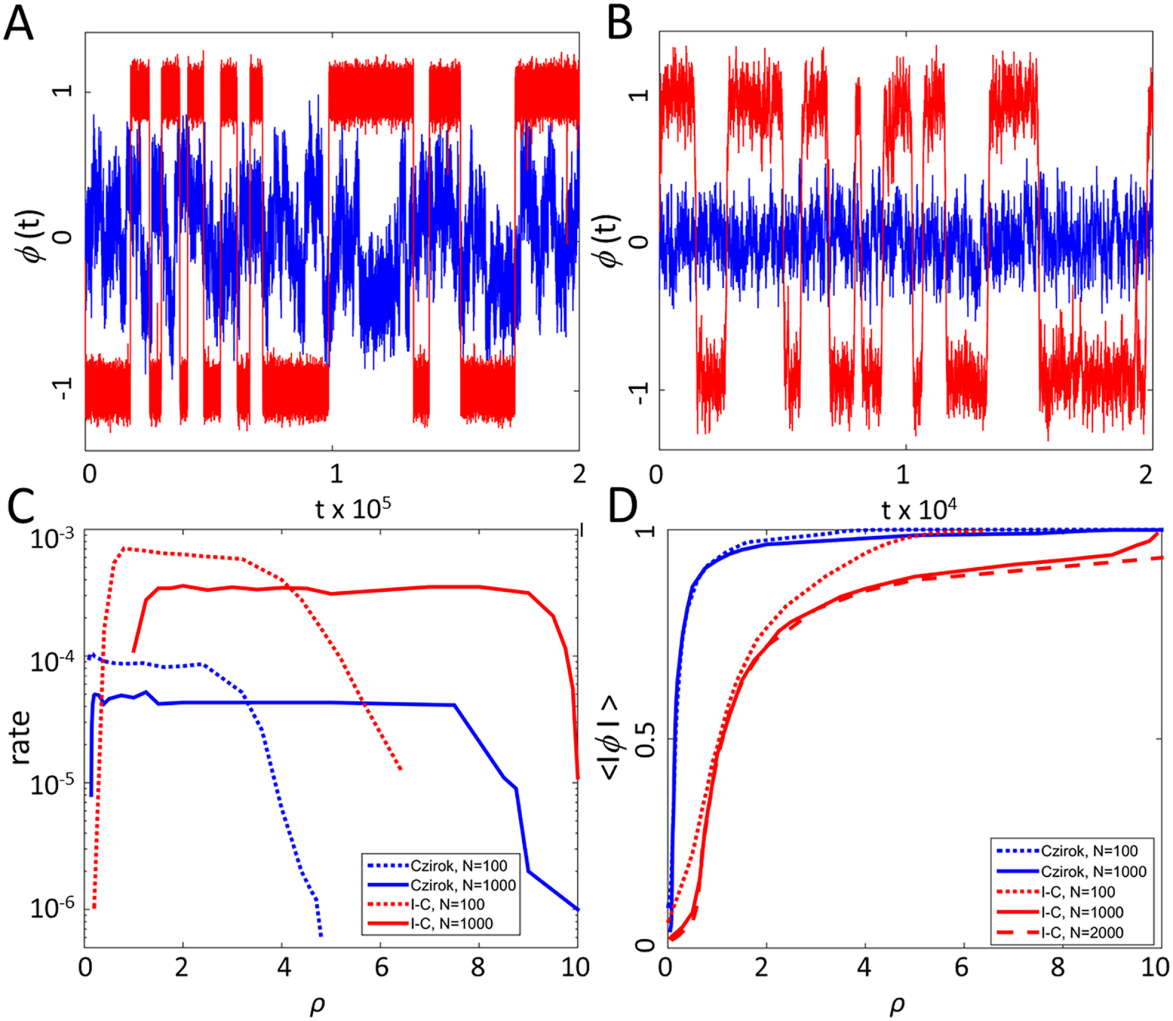
(A) Sample trajectories for the Czirók model. Blue curve: *ρ* = 0.1. Red curve: *ρ* = 3.2. (B) Sample trajectories with individual-choice (I-C) interactions. Blue curve: *ρ* = 0.2. Red curve: *ρ* = 3.2. Note that the time scale between two models differs by a factor of ten. (C) Mean transition rates as a function of *ρ*. (D) The average order parameter as a function of *ρ*.

Fig 2A depicts two sample trajectories of *ϕ*(*t*) at different concentrations of *ρ* = *N* / *L*. In the following, simulation results are presented with the parameters *N* = 100, *η* = 2, Δ = 1, and *v* = 0.1, unless specified otherwise. At low concentrations, the system is disordered and *ϕ*(*t*) fluctuates around zero. However, at higher concentrations, *ϕ*(*t*) fluctuates around one of two metastable states located at +1 and -1, with occasional rapid transitions between them. This suggests that, when order is high, the system admits a coarse-grained description as a two-state continuous time Markov chain (CTMC). S1A Fig shows the distribution of waiting times between transitions, which is approximately exponential, as expected for a CTMC. In simulations, metastable states were defined using a cutoff for *ϕ*(*t*), *A*_+_ = {*ϕ*(*t*)>*p*}, and *A*_-_ = {*ϕ*(*t*)< -*p*}, where *p* = 0.7. Fig 2C shows the average transition rate between the two metastable states for fixed noise *η* = 2 and varying *ρ* (see also S2 Fig). Interestingly, the switching rate shows a plateau for a wide range of concentrations. The length of the plateau seems to grow with *N*, suggesting a limiting equilibrium rate at the thermodynamic limit (*N*→∞ at constant *ρ* and *η*), which is the biologically relevant limit for natural large locust swarms.

The main significance of the Czirók model and its 2D predecessor [74] is the understanding that the two types of behaviors—fluctuating around *ϕ* = 0 or *ϕ* = ±1—can be thought of as phases in the sense of statistical physics. Consider the average order parameter

ϕ=〈|ϕ(t)|〉,

where brackets denote averaging over all time steps and initial conditions. Fig 2D shows the average order parameter in the Czirók model (blue lines) as a function of *ρ* for fixed noise *η*. The figure suggests that for large *N*, the curve becomes singular at a critical density *ρ*_*c*_. In statistical physics, this type of behavior is called a phase transition (not to be confused with the solitarious-gregarious transition discussed above) [62,64,65,74]. If the function *ϕ*(*ρ*) is discontinuous at *ρ*_*c*_, then the transition is a first order transition, similar to the solid/liquid/gas transition in solid state physics. However, if *ϕ*(*ρ*) is continuous at *ρ*_*c*_, but not differentiable, then the transition is called a second order transition, similar to the transition from a paramagnet to a ferromagnet in iron (a ferromagnet can remain magnetized after an external magnetic field is switched off, while a paramagnet cannot). Similar behavior is observed at constant *ρ* and variable *η*, indicating that *η* is analogues to temperature. See [76] for a discussion of temperature in Viscek-type models. The type of transition, critical concentrations, critical noise, and exponents in SPP models are studied numerically [65,72,74,77].

The significance of the observation that the Czirók model undergoes a phase transition is in understanding that order and disorder can be characterized as two distinct phases. Switching between the phases requires some external change in the system parameters, such as concentration or the amount of noise.

#### Individual-choice model

O'Loan and Evans [78] introduced an assumption that at every simulation step Δ*t*, animals make a decision as to whether to persist with their own velocity *u*_*i*_ or to align with the local order <*u*_*i*_(*t*)>. This is implemented as a random choice: at every time step, a particle retains its velocity with probability *α*. Otherwise, it aligns with neighbors according to the interaction rule described in Eq 2.

Fig 2B shows two typical trajectories at low and high concentrations in the individual-choice model with an interaction rule, following O'Loan and Evans. Although *ϕ*(*t*) is qualitatively similar to that of Czirók et al. [72], transition rates are higher by a factor of 10 (see Fig 2D). Note that neither Czirók et al. [72] nor O'Loan and Evans [78], which predate the experimental results of Buhl et al. [58], engaged with transition rates.

While the individual-choice model does present a phase transition, it does so in a different range of parameters. Fig 2C shows the average order parameter as a function of *ρ* for fixed noise, *η* = 2. The change from disordered (small *ϕ*) to ordered (large *ϕ*) behavior is smooth, and it is not clear whether a phase transition exists.

The view of the system dynamics as a sequence of discrete decisions is also at the heart of our model, discussed in the section “Individual pause-and-go movement” below. See also [79] for a general discussion of the importance of the decision-making process in collective movement.

#### Internal versus external noise

The Gaussian white noise in Eq 1 can be considered as an external source of noise since it is explicitly added to the equations of motion. In particular, if *σ* = 0, then the dynamics is deterministic. On the other hand, the random individual-choice noted above is best described as internal noise. In [78], two other sources of internal noise are studied:

- Asynchronous updating: At every simulation step, *N* particles are chosen at random (with repetitions) and updated sequentially.
- Alignment with a single neighboring particle chosen randomly instead of the local average <*u*(*t*)>_*i*.

See also [77,80,81]. Neither mechanism seems to have a significant effect on the dynamics.

#### Placing more emphasis on the individual animal

Motivated by their laboratory experiments with marching locusts in a circular arena, Buhl et al. [58] suggested a modified version of previous models and introduced the biologically reasonable assumption that animals weight their own direction separately to that of their conspecifics. Accordingly, the dynamics for *u*_*i*_ in Eq 2 is taken to be

$$u_{i}(t+1)=\alpha u_{i}+(1-\alpha )G\left({\langle u(t)\rangle }_{i-}\right)+\xi_{i}(t),$$ (3)

where 0≤*α*≤1 describes the propensity of an individual to persist with its own velocity rather than adapt to the swarm (in simulations, they set *α* = 0.66), and

$${\langle u(t)\rangle }_{i-}=\{\begin{array}{cc} \frac{1}{n_{i}(t)-1}\sum_{j\in A_{i}(t),j\neq i}u_{j}(t) & n_{i}(t)\geq 2\\ 0 & n_{i}(t)=1 \end{array}$$ (4)

is the local average direction around particle *i*, excluding *i* itself. See also [82] for a later 2D model.

The role of the parameter *α* can be described from two points of view. The first is an averaged (and, therefore, deterministic) implementation of the intrinsic noise suggested by O'Loan and Evans [78]. Indeed, for large swarms (*N*>100), the average order parameter in the two models [58,78] is practically the same, while transition rates with Buhl are typically higher (S3 Fig). On the other hand, the introduction of the parameter *α* is similar to using a smaller step size Δ*t* and rescaling noise. Indeed, taking *α* = 1-Δ*t*, yields an equation of the form of Eq 2 with a rescaled parameter *η*.

Removing the effect of particle *i* itself from the local average 〈*u*(*t*)〉_*i*_ has a subtle effect on the dynamics, which is similar to adding a friction term with a coefficient that decreases with the number of neighbors. In particular, the dynamics of an independent particle in the absence of neighbors changes drastically. Under Eq 2, the dimensionless velocity of an independent particle satisfies ${\dot{u}}_{i}=-(u_{i}\mp 1)/2+\sigma {\dot{W}}_{i}$, which is an Ornstein-Uhlenbeck process. The equilibrium distribution of the process has a mean of +1 or -1 and a variance *σ* ^2^. On the other hand, using 〈*u*(*t*)〉_*i-*_ instead of 〈*u*(*t*)〉_*i*_ in Eq 2, a particle with no neighbors satisfies ${\dot{u}}_{i}=-u_{i}+\sigma {\dot{W}}_{i}$. This is again an Ornstein-Uhlenbeck process, with the same equilibrium variance, but zero mean. In other words, the velocity fluctuates around zero. In this respect, particles are not “self-propelled.”

As explained above, the main purpose of the Buhl model was to interpret the experimental results obtained with locusts in a circular arena. Accordingly, it considers mostly systems with a relatively small number of particles (*N*≤100) in an arena with a fixed length (*L* = const). In this setting, it is found that the rate of switching between *A*_+_ and *A*_-_ decays exponentially with the number of particles *N* (compare S1B Fig with the relatively constant transition rate at large *N* depicted in Fig 2D). In addition, switching rates depend significantly on the influence of conspecifics on the individual, which is specified by the parameter *α* (S1C Fig). All other parameters correspond to the experimental conditions, as described by Buhl et al. [58]. The dependency on *α* is non-monotonic and varies by three orders of magnitude. In the Czirók model [72], rates are at least 4–5 orders of magnitude smaller than in the Buhl model. In this respect, the Czirók model fails to describe the dynamics observed in the experiments.

### Individual pause-and-go movement

Following their own experimental observations of locusts marching in a ring-shaped arena, Ariel et al. [59] suggested a modified model, based on the biological paradigm that animal decisions constitute a sequence of discrete events—when to stop, walk, or turn, rather than a continuous process. The main observation underlying the model is that the locusts follow an intermittent motion pattern that is composed of interchanging walking and standing periods (see also [23,24]; details in the above section, “Locust swarming and coordinated movement”). Pause-and-go motion was previously observed by [83] in an arena housing a single locust. In the Ariel et al. pause-and-go model [59], a particle is either standing (*v* = 0) or moving. Standing particles "do not participate in the game," i.e., they are ignored by moving particles and are not taken into account in calculating local averages. Similarly, the local average velocity and the order parameter are defined in terms of only moving particles, for example,

$${\langle u(t)\rangle }_{i,\text{moving}}=\{\begin{array}{cc} \frac{1}{n_{i,\text{moving}}(t)}\sum_{j\in A_{i,\text{moving}}(t)}u_{j}(t) & n_{i,\text{moving}}(t)\geq 1\\ 0 & n_{i,\text{moving}}(t)=0, \end{array}$$

and

$$\phi_{\text{moving}}(t)=\frac{1}{N_{\text{moving}}(t)}\sum_{i\text{ is moving}}u_{i}(t).$$

Ariel et al. [59] studied a pause-and-go model based on first-principle interactions as obtained from experiments. Here, we present a modified version of the model, which is easier to compare with the models discussed above:

1. A walking particle moves with a fixed velocity *vu*_*i*_ for a duration that is exponentially distributed with rate *k*_walk_.
2. A standing particle starts moving with rate *k*_stand_ that depends on the number of walking neighbors. The function *k*_stand_(*n*_i,moving_) is increasing in *n*_i,moving_, i.e., local movement increases the probability of standing animals to start walking. For simplicity, we take *k*_stand_(*n*_i,moving_(*t*)) to be a step function that jumps from a low rate *k*_stand,0_ to a higher one *k*_stand,1_ if *n*_i,moving_(*t*) is above a threshold *n*_c,moving_.

When a particle starts walking (i.e., changes from a standing to a walking state) it has a probability *α* to align with the local order among moving individuals, plus the usual noise term, $u_{i}(t+\Delta t)=u_{i}(t)+\left[G\left({\langle u(t)\rangle }_{i,\text{moving}}\right)-u_{i}(t)\right]\Delta t+\sqrt{\Delta t}\xi_{i}(t)$. However, the probability *α* depends on the local order 〈*u*(*t*)〉_i,moving_. For simplicity, we assume that \*alpha*(*x*) is a linear, increasing function. This dependence implies that animals have a higher tendency to align with ordered crowds than with disordered ones.

S4 Fig shows the global order parameter as a function of concentration for the pause-and-go model described above. The model suggests some qualitative predictions on the global properties or coarse-grained dynamics of the swarm, as discussed in the section “Coarse-Graining and Macroscopic Observables” below.

### Three-zone models

One of the first modeling approaches for collective motion, proposed in the early 1980s by Akoi [84] and independently by Reynolds [85], is the widely used three-zone, or Avoidance–Alignment–Attraction (AAA), model. The model, popular with biologists for its intuitive assumptions, but less so with theoreticians for its difficult analysis, assumes that the interaction between conspecifics can be divided into the zones defined by three characteristic lengths, as follows.

- Individuals are repelled from conspecifics that are closer than a distance *r*_1_.
- If no conspecifics are present up to distance *r*_1_, then an individual aligns its movement direction with conspecifics that are closer than a distance *r*_2_>*r*_1_.
- If no conspecifics are present up to distance *r*_2_, then an individual is attracted to conspecifics that are closer than a distance Δ>*r*_2_.

A 1D version of the model was studied in the context of locust swarming by Bode et al. [80]. The algorithmic implementation of their model, which assumes asynchronous dynamics, is as follows. For every time step Δ*t* repeat *N* times:

1. Choose an individual *i* at random.
2. If *i* has neighbors (up to distance Δ), choose a neighbor *k* at random and update *u*_*i*_ according to
  $$u_{i}=\{\begin{array}{cc} G(u_{k}) & |x_{i}-x_{j}|\leq r_{2}\\ G\left(\text{sgn}(x_{k}-x_{i})\left(\frac{\left|x_{k}-x_{i}\right|}{\Delta -r_{2}}+1\right)\right) & r_{2}<|x_{i}-x_{j}|\leq \Delta \end{array}.$$
3. Advance: *x*_*i*_ = *x*_*i*_+*vu*_*i*_Δ*t*.

Note that the model does not include avoidance interactions, which, as far as locusts in a circular arena are concerned, do not make sense in 1D. Bode et al. [80] analyzed the dynamics under their model using coarse-grained variables, as described in the section “Coarse-Graining and Macroscopic Observables” below.

### Escape and pursuit

Recently, a new paradigm that strives to give a comprehensive answer to several aspects of the locust phenomenon focused on cannibalism as a dominant feature of locust behavior and interactions ([33–35], and see 2.2 above). Building on this idea, Romanczuk et al. [63] suggested a modeling approach termed “escape and pursuit” (E&P). In this model, animals are continuously escaping from conspecifics behind them in order to avoid being eaten, while at the same time pursuing other animals in front, attempting to eat them.

The E&P model is a 2D SPP model. As an extension of the approaches described in the section “Locusts as self-propelled particles,” above, neighbors of a focal particle are categorized into four types: either at the front or behind, and either approaching or receding. Here, conspecifics that are either approaching from the front or receding from the back are ignored. Each of the two remaining types is averaged and weighted separately.

To be precise, the model assumes that individuals follow Langevin dynamics of the form

$$\begin{array}{l} \dot{x}=vu_{i}\\ {\dot{u}}_{i}=-\gamma u_{i}+F_{i}^{S}+\sqrt{2D}{\dot{W}}_{t}, \end{array}$$

where *γ* and *D* are the friction and diffusion coefficients, respectively, and $F_{i}^{S}$ describes the effective social force on particle *i*, which is given by

$$\begin{array}{l} F_{i}^{S}=f_{i}^{e}+f_{i}^{p}\\ f_{i}^{k}=\frac{\chi_{k}}{N_{k}}\sum_{j}\Delta u_{ji}H(R-r_{ji})H(s_{k}u_{i}{\hat{r}}_{ji})H(s_{k}u_{ji}{\hat{r}}_{ji}). \end{array}$$

Here, *k* = *e*,*p* is the effective escape (*e*) and pursuit (*p*) response. The heaviside function is denoted by *H*(·), and $\Delta u_{ji}=(u_{ji}{\hat{r}}_{ji})r_{ji}$ is the relative dimensionless velocity of particle *j* with respect to *i*, where *u*_*ji*_ = *u*_*j*_-*u*_*i*_, *r*_*ji*_ = *r*_*j*_-*r*_*i*_, and ${\hat{r}}_{ji}=r_{ji}/|r_{ji}|$. In addition, *χ*_*k*_ are interaction strengths, and *N*_*k*_ the number of particles *i* is pursuing or escaping. See [63] for details.

Unfortunately, a 1D version of the E&P model is problematic because, depending only on the initial condition, animals split into CW and CCW moving groups that do not interact. This observation is not consistent with our own experimental observations [59] indicating that one of the principle sources of synchronization in marching nymphs is that of animals turning to join a crowd moving in the opposite direction.

While offering a very attractive mechanism, the E&P model is somewhat inconsistent with various biological observations. As noted above, individuals in a marching band will very often stop for periods of various durations (see also [23,24]); when encountering a standing hopper, the marching ones will ignore it (Fig 1D). Furthermore, as noted in the section “Locust swarming and coordinated movement” above, locust marching behavior is mostly very different from escape or aversive reactions [27], which usually involve escape jumps [26,28]. Undisturbed marching desert locusts are very rarely seen hopping or jumping [29]. Also inconsistent with E&P is the behavior of hoppers at the front and side edges (e.g., the tendency of leading locusts to slow down or even turn around to join the bulk of the swarm [23,24,27,30]; detailed in the section “Locust swarming and coordinated movement”). According to the E&P model, locusts will chase receding conspecifics in front of them, escape conspecifics approaching from behind, and ignore others (approaching from the front or receding at the back). Therefore, under the rules of E&P, a nymph moving beyond the front of the swarm will not turn around but, rather, keep escaping the approaching swarm at its back.

Buhl et al. [86] reported their findings in a study of the Australian plague locust specifically aimed at analysing the spatial distribution of locusts within naturally occurring locust bands and inferring on the interactions within the band. These authors reported a tendency for locusts to interact with neighbours all around them, rather than a bias toward pursuing individuals ahead or escaping from the ones following behind. The E&P model may account for the collective behavior of locust nymphs under certain conditions and circumstances, in accordance with the context- and time-dependent role of cannibalism suggested in the section “Locust swarming and coordinated movement.”

### Interim summary

Most of the models presented above share a common assumption regarding the animal’s calculation of a local average (or weighted average) that serves as the main influence of conspecifics on the individual. From a biological perspective, this assumption is not realistic, as insects and other animals are often more sensitive to extremal statistics, such as abrupt or unexpected changes (in the behavior of others or in the environment). In this sense, the pause-and-go model, in which the probability of an animal to start marching depends on the visual flux around it, more precisely on the number of walking neighbors exceeding a threshold, may be more realistic.

The next section further compares the different models in terms of the macroscopic dynamics of the entire swarm.

## Coarse-Graining and Macroscopic Observables

In order to quantify and analyze metastability in the dynamics of the order parameter, Yates et al. [75] suggested fitting the dynamics to a diffusion equation of the form

$${\dot{\phi }}_{t}=F(\phi )+\sqrt{2D(\phi )}{\dot{W}}_{t},$$ (5)

where, *F*(*ϕ*) and *D*(*ϕ*) are effective drift and diffusion functions. See also [59,70,71,80]. Similar derivations can be obtained by fitting to an effective Fokker-Plank equation for the time-dependent probability density for *ϕ*, *f*(*ϕ*,*t*), which is also the Kolmogorov forward equation for Eq 5,

$$\frac{\partial f}{\partial t}=\frac{\partial^{2}\left(D(\phi )f\right)}{\partial \phi^{2}}-\frac{\partial \left(F(\phi )f\right)}{\partial \phi }.$$

The effective drift and diffusion functions can be approximated from the dynamics as

$$\begin{array}{l} F(\phi )=\frac{1}{\Delta t}{\langle \phi_{t+\Delta t}-\phi_{t}\rangle }_{\phi }\\ D(\phi )=\frac{1}{2\Delta t}{\text{Var}}_{\phi }\left[{\left(\phi_{t+\Delta t}-\phi_{t}\right)}^{2}\right]=\frac{1}{2\Delta t}\left[{\langle \phi_{t+\Delta t}^{2}\rangle }_{\phi }-{\langle \phi_{t+\Delta t}\rangle }_{\phi }^{2}\right], \end{array}$$ (6)

where brackets denote averaging over all instances in which the order parameter is within some range around *ϕ* and Δ*t* is a small time segment. The choice of Δ*t* is critical and is closely related to the different time scales that appear in the dynamics. In [70,75,80], the second moment of *ϕ*_t+Δt_-*ϕ*_*t*_ is used instead of the variance. In the limit of Δ*t*→0, 〈*ϕ*_t+Δt_-*ϕ*_*t*_〉_*ϕ*_ is of order Δ*t*, and the two expressions are the same. However, as discussed below, this is not necessarily the biologically relevant limit.

The main advantage of approximating the dynamics of *ϕ*_*t*_ as a diffusion process lies in providing a low-dimensional, coarse-grained description, which may assist us in gaining insight into the dynamics of the entire swarm. Accordingly, the evolution of *ϕ*_*t*_ occurs on a time scale comparable to experiments—from several minutes to hours. On this relatively long time scale, locusts can circle the arena hundreds of times, which means that during the experimental time, the coarse-grained dynamics includes both spatial averaging over all animals and temporal averaging. This suggests that Δ*t* should be on the scale of *L* / *v*~1min. A detailed discussion on the choice of Δ*t*, including a thorough comparison between Eq 6 and the methods presented in [70,75,80], is included in the Supporting Information.

The coarse-grained analysis offers an accessible interpretation of the macroscopic dynamics of the swarm. The effective drift function describes the average rate of change in the order parameter. A positive value of *F*(*ϕ*) implies that when the order parameter is around the value *ϕ*, then it will (on average) increase. Similarly, negative *F*(*ϕ*) implies that the order will decrease. Thus, zeros of *F* indicate fixed points for the average. Fixed points can be categorized as either stable or unstable, depending on whether by changing *ϕ* by a small value, the order parameter will tend to go back to the fixed point or not. In other words, close to a stable point ϕ*, if *ϕ* increases (ϕ>ϕ*), *F*(*ϕ*) is negative, which means that on average, *ϕ* will decrease back to ϕ*. On the other hand, if *ϕ* decreases (ϕ<ϕ*), *F*(*ϕ*) is positive and *ϕ* will increase. Therefore, the metastable states of the system are characterized by stable zeros of *F*(*ϕ*). Similarly, the effective diffusion function *D*(*ϕ*) roughly describes the amount of noise in the system, where large *D*(*ϕ*) implies high noise.

**Fig 3:**
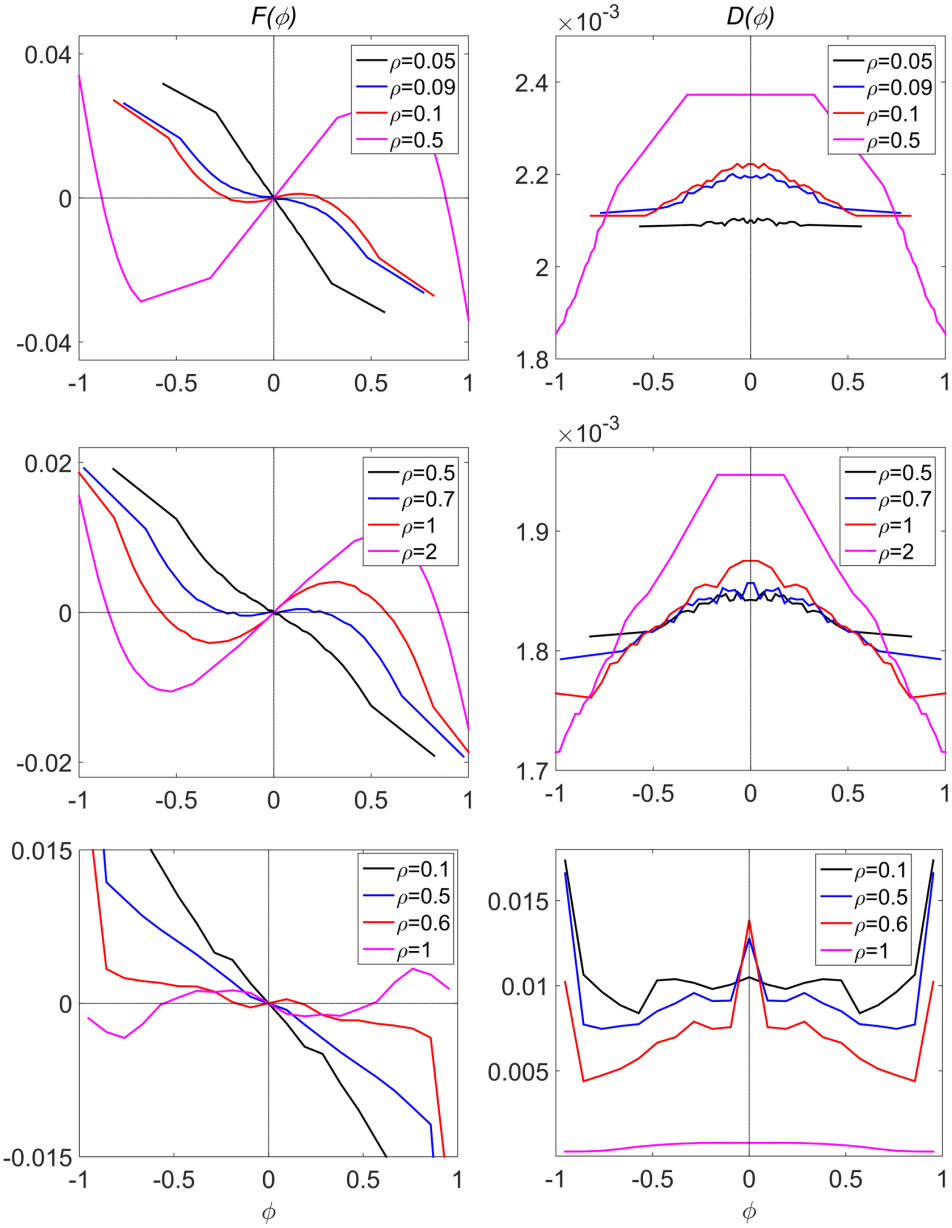
Coarse grained dynamics. Left: the effective drift *F*(*ϕ*). Right: The effective diffusion *D*(*ϕ*). Top: for Czirók model. Middle: Buhl model. Bottom: Pause-and-go model. In both the Czirók and Buhl models, depending on concentration, either *ϕ* = 0 or *ϕ = ~±*1 are stable, but not both. However, in the pause-and-go model, all three states are stable at sufficiently high concentrations.

Fig 3 compares *F*(*ϕ*) and *D*(*ϕ*) as obtained in simulations of the Czirók, Buhl, and pause-and-go models at different particle densities. At low concentrations, the only stable state for all three models is disordered, *ϕ>ϕ* = 0. However, at higher concentrations, the pause-and-go model behaves qualitatively differently to the other two. In both the Czirók and Buhl models, the system has two stable fixed points close to *ϕ* = ±1, while *ϕ* = 0 is unstable. In contrast, with pause-and-go, all three states are stable, indicating that the disordered state is also a metastable state of the system. However, the residence time in the disordered state is significantly lower. All models agree that noise, as described by the effective diffusion function *D*(*ϕ*), has a local maximum at *ϕ* = 0. However, the pause-and-go model also shows large noise for |*ϕ|>*1. Compare also with experimental results adapted from [59], depicted in S5 Fig. Note the difference in the effective diffusion compared to the results of [75] due to a different choice of Δ*t*. See Supporting Information for details.

## Some Further Modeling Challenges: Continuous Models

As discussed above, continuous models are particularly useful in describing global properties of large, many-particle systems. To the best of our knowledge, only three such models have been developed, none of which relate to the phenomenon of marching locusts. The first model, suggested by Edelstein-Keshet et al. [87], concerns flying locust swarms. The other two models, Feistel and Feistel [88] and Topaz et al. [89], focus on the transformation from the solitary to the gregarious behavioral phase.

### Modeling adult locust flying swarms

The model of Edelstein-Keshet et al. [87] studies the swarming behavior of locusts, focusing on the long-distance migratory swarms of flying adults. The main objective of this work was to investigate the issue of maintaining swarm cohesion; specifically, to find traveling wave solutions, which are particular solutions that have a fixed shape and move at constant speed. The model is given in terms of partial differential equations (PDEs) describing the time evolution of the continuous density of flying locusts, *f*(*x*,*t*), and stationary locusts (those on the ground), *s*(*x*,*t*). In this respect, the swarm exhibits intermittent motion reminiscent of the pause-and-go model. However, the latter includes a mechanism for turning and choice of direction that is missing here. In addition, there are no periodic boundary conditions. For simplicity, the swarm is considered as 1D. The dynamics is given by

$$\begin{array}{l} \frac{\partial s}{\partial t}=-R(s,f)s+G(s,f)f\\ \frac{\partial f}{\partial t}=Df_{xx}-Uf_{x}+R(s,f)s-G(s,f)f, \end{array}$$

where, *R*(*s*,*t*) is the take-off rate, *G*(*s*,*f*) the landing rate, *D* describes random motion (when flying), and *U* describes the flying velocity (wind speed and self-propulsion). More elaborate models that take into account additional effects, such as dependence of *D* and *U* on the local densities, slow motion on the ground, and density-dependent turning, are also considered. The main conclusion of the paper is that traveling waves, representing cohesively moving swarms, cannot exist unless non-local interactions, i.e., global interaction between animals within the entire swarm, are allowed. Even then, “traveling wave solutions seem to bear some unrealistic properties.” See [87] for details.

### Modeling locust phase transformation

Feistel and Feistel [88] approach the question of locust phase polyphenism and the concentration-dependent transition from the point of view of statistical physics. Assuming a homogenous, well-mixed population, they consider a biologically realistic mean-field model that describes the time evolution of four parameters: (i) External mechanical stress that increases production of (ii) a stress hormone. The latter affects individual behavior by reducing the (iii) average local concentration, increasing (iv) the rate of collective interactions. Increased collective interaction increases the external mechanical stress, thus creating a positive feedback loop. It is shown that, depending on model parameters, the dynamics undergoes a bifurcation, and the population can be classified into three phases: solitary, gregarious, and unstable. Transitions are analogous to either first- or second- order phase transitions in statistical physical, depending on parameters, such as the stress hormone production rate.

The model of Topaz et al. [89] studies the phenomenon of locust outbreak, which is characterized by rapid large-scale transitions between scattered populations of solitary animals to densely-packed gregarious swarms (see also [90]). The Topaz model is given in terms of integro-differential equations that describe the time evolution of a two-dimensional density of solitary animals, *s*(*x*,*t*), moving at velocity *v*_*s*_(*x*,*t*), and the density of gregarious animals, *g*(*x*,*t*), moving at velocity *v*_*g*_(*x*,*t*). Densities satisfy the continuity equation (conservation of mass)

$$\begin{array}{l} \frac{\partial s}{\partial t}+\nabla \cdot (v_{s}s)=-k_{s\to g}(s+g)s+k_{g\to s}(s+g)g\\ \frac{\partial g}{\partial t}+\nabla \cdot (v_{g}g)=k_{s\to g}(s+g)s-k_{g\to s}(s+g)g, \end{array}$$

where *k*_*s→g*_(*ρ*) and *k*_*g→s*_(*ρ*) are the rate of transition from solitary to gregarious and vice versa, at a total local concentration *ρ* = *s*+*g*. It is assumed that gregarious animals are attracted to conspecifics while solitary animals are repelled. The strength of the interaction decreases exponentially with distance,

$$\begin{array}{l} v_{k}=-\nabla (Q_{k}*(s+t))\\ Q_{k}(x-x\prime )=R_{k+}e^{-|x-x^{\prime }|/r_{k+}}-R_{k-}e^{-|x-x^{\prime }|/r_{k-}}, \end{array}$$

where * denotes convolution and *R*_k±_,*r*_k±_ are parameters, *k* = *g* or *s*. Numerical solutions as well as stability analysis reveal conditions for outbreaks.

It should be noted that our current understanding of questions related to solitary locust ecology and population dynamics, such as competition over resources, sexual selection and mate finding, habitat range, displacement, and more, is very limited, as most research focus has naturally been placed on the gregarious phase.

The work of Topaz et al. [89] strongly suggests the need to integrate the complex effects of density, as well as the dynamics of phase transformation, in the models of marching behavior (“Modeling Marching Locust Nymphs,” above). This model stands out in making an attempt to bridge different scales, from the large scale dynamics accompanying the emergence of swarms of gregarious locusts from scattered solitary populations, to the more local scale of the mechanisms inducing coordinated motion of the swarms.

## The Evolution of the Locust Phenomenon

Although it is beyond the scope of the present review, it should be added here that, to the best of our knowledge, there have been surprisingly few attempts at explaining the very puzzling question of the evolution of phase transformation and swarming within the group comprising grasshoppers and locusts, by way of mathematical or theoretical modeling. As mentioned earlier, our understanding of the evolution of locust phase polyphenism is hindered by the complexity of the phenomenon: (i) Locusts do not necessarily form a monophyletic group (a group containing an ancestor and all of its direct descendants), but rather belong to at least six separate subfamilies [8]. Hence, locusts have independently evolved more than once, or several times. (ii) Locust phase polyphenism is a composite character, consisting of numerous density-dependent phenotypically plastic traits [6,7,8], which, most importantly, may have followed different evolutionary trajectories. (iii) Different locust species (and, to an extent, other grasshoppers as well) differ in their ability to express the phenomenon in its entirety—i.e., in the magnitude of their response to changes in population density [9,10]. Therefore, a single unifying explanation or theory is unlikely.

Nonetheless, several attempts in this direction have been reported, typically extending the perspective beyond locusts per se, for example, by looking at polyphenism and phenotypic plasticity in general (in insects and beyond); i.e., explaining the functional relationship between genetic, morphological, physiological, and environmental variables, and how changes may accumulate and stabilize over generations. Beldade et al. [91] include locusts in describing mechanisms inducing adaptive developmental plasticity, from the genetic and molecular to the environmental. Others have used a more mathematical framework to explain the evolution of dispersal and migration in nature. In a recent example, Gueijman et al. [92] demonstrate that stable variations in the tendency to disperse (such as the difference between gregarious and solitary locusts) can evolve by introducing a model of Fitness-Associated Dispersal, or FAD (see [92] and references therein for a discussion of different models explaining the evolution of plasticity in dispersal). In another example, Berdahl et al. [93] explore the interactions of dispersal and local adaptation as major drivers of population structure, finding that environmental conditions may lead to either a polymorphic population or a monomorphic population of highly dispersing individuals. Moving to the evolution of collective migration, Guttal and Couzin [94] offer an individual-based, spatially explicit model demonstrating that collective migratory strategies evolve under a wide range of ecological scenarios, and introducing the importance of social interactions.

Two theoretical studies, by Reynolds et al. [95] and Guttal et al. [96], attempt to provide a plausible model for the evolution of polyphenism in locusts. The first focuses on the interactions between locusts and their predators. Loosely speaking, assume that locusts live within isolated food patches of variable size. Also assume that a patch with a sufficiently high concentration of locusts will attract a predator. The network of patches is modeled as a graph in which different patches are nodes. Nodes connected by an edge are those between which the predator can traverse while still finding locusts in sufficiently high abundance. The question thus becomes: is it better for locusts to disperse between patches or to aggregate in a small area? The answer, according to Reynolds et al. [95], depends on locust density. If the number of locusts is low, it is better for them to disperse between patches (a solitary behavior), as most feeding locations will then have a low concentration, making them unfavorable to predators. As the number of locusts increases, more and more locations will become favorable to predators. Furthermore, percolation theory predicts that at sufficiently high concentrations, a giant (or infinite) cluster appears, in which predators will have an infinite connected network of favorable patches. At this stage, it is advantageous for the locusts to aggregate into a smaller number of highly populated patches (a gregarious behavior), in order to break the connectivity of the giant cluster. We find that the main advantage of this model lies in its simplicity. However, it ignores the collective motion phenomenon, which, as far as locusts are concerned, is a dominant feature of the gregarious behavior.

The second approach, by Guttal et al. [96], is based on the suggested instrumental role of cannibalism in locust marching behavior, reviewed in the section “Escape and pursuit.” Using an individual-based evolutionary model, they suggest that cannibalism could have been a key factor in the evolution of behavioral-phase polyphenism in locusts. Accordingly, the authors suggest an E&P model in which interactions are updated using an evolutionary algorithm. The main result is that, at low locust concentrations, the insects can minimize the risk associated with cannibalistic interactions by keeping their distance from conspecifics. However, at higher concentrations, this strategy is no longer favorable, and it is preferable to stay in an ordered, cohesively moving swarm. These results can be interpreted as a combination of the order-disorder phase transition in the SNM of Viscek et al. [74], and the solitary–gregarious phase transition described by Feistel and Feistel [88].

## Concluding Remarks

Modeling and understanding locust swarming has a dual role, or presents two approaches. The first views locusts as a quintessential example of animal collective motion. As such, due to major advances in experimental and animal tracking technologies, it allows the testing and evaluation of theories regarding the onset and maintenance of order and synchronization in moving swarms. Indeed, most of the models presented above take this approach, as they aim at abstraction rather than analysis and prediction of natural swarm dynamics. These models correspond to the general literature on collective motion, at the risk of failing to capture features unique to the locust swarms.

Locusts have much to offer with respect to our general understanding of coordinated movement in nature. Similarly, we can take advantage of successful modeling efforts in other organisms in order to explain the locust phenomena. Indeed, one of the major remaining challenges in the growing field of collective animal movement lies in relating the similarities and differences between the mechanistic and behavioral modes of motion of different organisms to the observed large-scale coordinated behavior they exhibit.

The second approach views locust swarming as a scientific problem of its own merit. From this more biological perspective, the main goal will be to use experiments in order to identify the principal interactions between animals and apply them in agent-based models. Our own work in [59] offers an initial step in deriving a simplified model based on first principle dynamics. In particular, all parameters used are fitted from experiments. The successful use of modeling efforts in describing and, moreover, in predicting the dynamics of real-life locust swarms crucially depends on taking into account many aspects and details of locust movement known to have a pivotal impact on their behavior: for example, the effect of temperature, the choice of direction, daily behavioral patterns, differences between marching and flight, etc.

The locust phenomenon comprises different spatial and temporal scales, ranging from the individual (centimeters and minutes) all the way to vast, natural swarms consisting of millions of insects (kilometers and hours). Currently, there is a gap between the individual-based approaches presented early in this review (the section “Modeling Marching Locust Nymphs”) and the later-discussed continuous models (the two last sections). A key direction for future work will be to employ realistic and biologically accurate models to drive coarse-grained continuous models in order to understand the dynamics of entire swarms. The discussion on time scales in the related section above (“Coarse-Graining and Macroscopic Observables”) is an example of the intricate manner in which the local and global dynamics are connected. From the point of view of spatial scales, additional experimental and theoretical work is needed in order to understand the effects of the environment, e.g., climate, topography, etc. The major effects of anthropogenic factors, before and mainly during outbreaks (e.g., the efficiency of pesticides in containing outbreaks), should also be considered. We should also attempt to include global population dynamics in addition to local ones—can we treat a species’ entire global population as one?. Some hints for future work may come from genetic analyses of divergence among locust populations (e.g., [97–99]). However, this direction still awaits interpretation by way of solid theoretical models.

Another significant gap in our understanding is related to the role of learning and memory-related mechanisms in the different aspects of locust behavior. Geva et al. [20] have made an important step in this direction by introducing a role for long-term memory in phase transformation. This should be extended to include a role for learning and memory in locust swarming and coordinated marching.

With the increasing accumulation of biological knowledge, it is becoming clear that only a combined-interdisciplinary, biological-theoretical effort can advance our understanding of the locust phenomenon in general and of locust collective movement in particular. There is great importance in performing further controlled empirical experiments to test the hypotheses generated by the various models and the predictions resulting from them.

## Supporting Information

S1 Text The time scale of the effective diffusion. (DOCX)

S1 Fig (A) Waiting time between switching orientations in the individual-choice model. The red curve shows the maximum likelihood fit to an exponential distribution, implying that waiting times are uncorrelated. (B) Transition rates for small swarms as a function of *N*. (C) Transition rates as a function of *α* with *N* = 10.
(TIF)

S2 Fig Mean transition rates as a function of *ρ* for different system sizes *N*. Left: Czirók model. Right: The individual choice model. Blue: *N* = 20, Black: *N* = 50, Magenta: *N* = 100, Green: *N* = 200, Orange: *N* = 1000, Red: *N* = 2000.
(TIF)

S3 Fig Comparison of random and deterministic weighing of individual preferences. In the model of O`Loan and Evans [78] (blue lines), an animal makes a random choice whether to persist with its own speed or align with conspecifics. In the model of Buhl et al. [58] (red lines), individuals weigh their own speed separately with all others. Taking all other interactions and parameters to be the same, the two models result in practically the same average order parameter (left plot). Transition rates (right plot) with Buhl’s model are typically higher. *N* = 1000; all other parameters are the same as in Fig 2.
(TIF)

S4 Fig The average order parameter as a function of concentration for the stop-and-go model. (all) The order parameter is calculated using all particles *ϕ* = 〈|(1 / *N*)Σ_*i*_ *u*_*i*_|〉. (moving) The order parameter is calculated using only moving particles *ϕ* = 〈(1 / *N*_moving_)Σ_i is moving_ *u*_*i*_〉.
(TIF)

S5 Fig The effective drift and diffusion as obtained in experiments. Adapted from [59]. The number of zeros of *F*(*ϕ*), corresponding to the metastable states of the system, is difficult to evaluate due to large statistical errors.
(TIF)

S6 Fig The effective drift (left) and diffusion (right) functions for the Czirók model using different time step Δ*t* = 1 (black), 0.2 (red), 0.04 (blue), and 0.008 (magenta). Solid line: using Eq 6. Dotted line: replacing the variance with the second moment.
(TIF)

S7 Fig Comparing the effective drift and diffusion functions using a single long (solid line, *T* = 10^5^) and many (10^5^) short (dotted line, *T* = Δ*t*) sample trajectories. Red: Δ*t* = 0.008, blue: Δ*t* = 1. With very small Δ*t*, the two sample methods yield practically the same effective diffusion but slightly different drift. However, with larger Δ*t*, both the drift and diffusion functions are different.
(TIF)
