# Supplementary material for: Locust Collective Motion and Its Modeling
Source: PLoS Comput Biol. 2015 Dec 10;11(12):e1004522. doi: 10.1371/journal.pcbi.1004522 (PMC4675544; doi:10.1371/journal.pcbi.1004522)
Supplement: S1 Text — (DOCX) [file pcbi.1004522.s001.docx]

**Supplementary Information**

The time scale of the effective diffusion

The Section **Course-graining and macroscopic observables** discusses coarse graining of the macroscopic dynamics and suggests fitting the dynamics to a diffusion equation of the form (5)

. (S1)

The effective drift and diffusion functions can then be approximated from the dynamics as

(S2)

and

(S3)

where brackets denote averaging over all instances in which the order parameter is within some range around and $\Delta t$ is a small time segment. The choice of $\Delta t$ is critical and may affect the observed effective parameters. Similar to the analysis in [75] (SM, section S.3), we analyze the errors introduced by (S2) by approximating (S1) as an Euler-Maruyama step,

, (S4)

where $\xi_{n}$ are independent standard normal random variables. Substituting into (S2) yields

.

In principle, vanishes, which validates (S2). However, due to finite sampling errors, the observed average of $\xi_{n}$ does not vanish exactly. Moreover, the error is inversely dependent on $\sqrt{\Delta t}$, which implies that decreasing $\Delta t$ requires increased sampling. Similarly, substituting (S4) into (S3) yields

.

Again, with infinite sampling, $\text{Var}\left[\xi_{n}\right]=1$, which validates (S3). Naturally, finite sampling introduces a statistical error. However, it does not depend on $\Delta t$.

In Yates et al [75], the authors evaluate the effective diffusion using the second moment of instead of the variance,

.

Substituting (S4) yields,

,

which converges to the correct diffusion in the limit of $\Delta t\to0$. However, in this case, the evaluation error is increasing with $\Delta t$ which motivated Yates et al [75] to use a very small step size, about $\Delta t=0.003$. Note that the time scale used in [75] is scaled to experimental time (see [75], their caption of Fig. 4). According to this scale, $\Delta t=0.003$corresponds to about 0.2 seconds in experiments.

S6 Fig. shows the effective drift and diffusion obtained with different time steps $\Delta t=0.008$, 0.04, 0.2 and 1. Using a sufficiently long trajectory ($10^{7}$ simulation steps), the drift is similar in all simulations. With larger step sizes, the shape of $F$ deviates, probably due to sampling errors. On the other hand, the diffusion varies considerably. Using a very small $\Delta t$, the diffusion is practically constant (full magenta line). In addition, $D$ (full lines) and $\tilde{D}$(dotted lines) are practically the same. This is the main results of Yates et al [75]. However, experimental results (for example, as depicted in S5 Fig.) show that the diffusion is non-monotonic and has a local maximum around . This difference motivates the introduction of a non-constant noise term into the Czirok model, rewriting (2) as

$$\begin{matrix} x_{i}(t+\Delta t)=x_{i}(t)+vu_{i}(t)\Delta t \\ u_{i}(t+\Delta t)=u_{i}(t)+\left[G\left(\left\langle u(t)\right\rangle_{i}\right)-u_{i}(t)\right]\Delta t+\sqrt{\Delta t}\sigma\left(\left\langle u(t)\right\rangle_{i}\right)\xi_{i}(t), \end{matrix}$$

Where $\sigma(\cdot)$ is takes to be a quadratic function with a maximum at zero. However, S6 Fig. shows that for larger values of $\Delta t$, the diffusion indeed becomes non-monotonic with a local maximum around , in accordance with experiments. In addition, the difference using the variance or the second moment becomes apparent as $\Delta t$ increases.

The main motivation for approximating the dynamics of as a diffusion process is in providing a low-dimensional, coarse-grained description of the macroscopic dynamics of the entire swarm. Accordingly, the dynamics of should describe the evolution of the order parameter on a time scale comparable to experiments – from several minutes to hours. On this relatively long time scales, locusts can go around the arena hundreds of times, which means that on the experimental time-scale, the coarse-grained dynamics should include both spatial averaging over all animals and temporal averaging. This suggests that $\Delta t$ should be of the scale of $L/v\sim1\min$. Accordingly, we argue that the $\Delta t\to0$is not necessarily the biologically relevant limit here and that larger values of $\Delta t$ are more appropriate.

Finally, [75] suggest replacing the averages in (S2) and (S3) which are taken with respect to long-time trajectories with many appropriately initialized short-time averages. S7 Fig. compares the effective drift and diffusion using long trajectories (solid lines) and many ($10^{5}$) short ones (dotted lines) using two different time steps - $\Delta t=0.008$ and $\Delta t=1$. Short trajectories were initialized with a uniform particle distribution and velocities that correspond to an given order parameter . We observe that both the effective drift and diffusion in both approaches are qualitatively the same. However, the numerical values are different expect for the diffusion obtained with very small $\Delta t$. The differences indicate that the effective equation for described by (S1) may not be closed in the sense that additional variables may be required for a complete coarse grained reduction of the macroscopic dynamics. For example, the typical distribution of particles may depend on the order parameter. Such effects are not taken into account in the approximation (S1).
