# Supplementary figures and images for: Locust Collective Motion and Its Modeling
Source: PLoS Comput Biol. 2015 Dec 10;11(12):e1004522. doi: 10.1371/journal.pcbi.1004522 (PMC4675544; doi:10.1371/journal.pcbi.1004522)

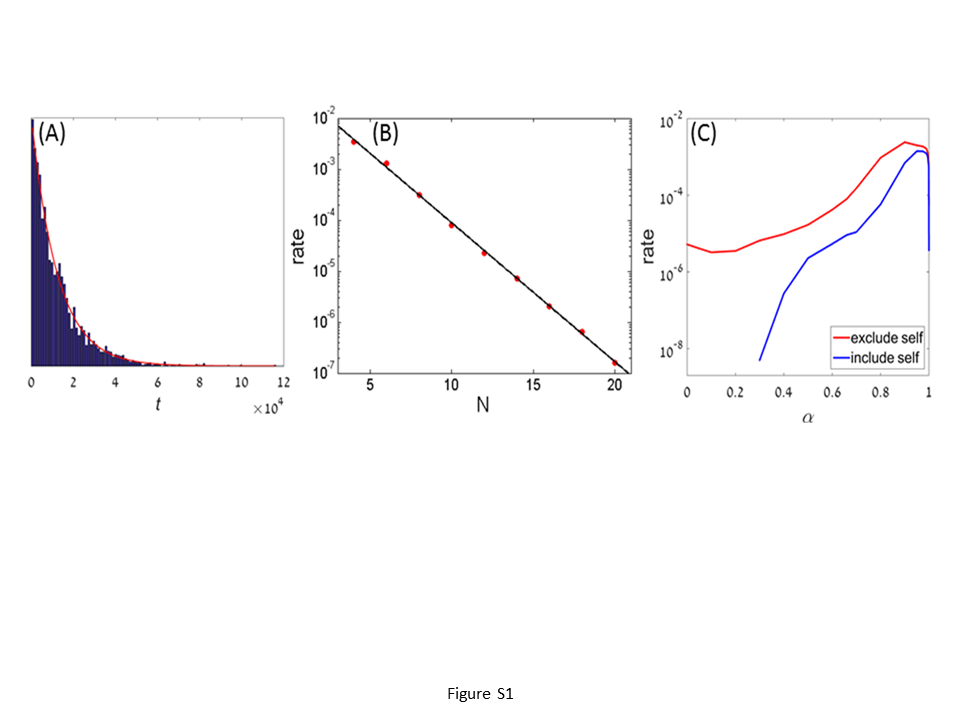

Supplement: S1 Fig — (A) Waiting time between switching orientations in the individual-choice model. The red curve shows the maximum likelihood fit to an exponential distribution, implying that waiting times are uncorrelated. (B) Transition rates for small swarms as a function of N. (C) Transition rates as a function of α with N = 10. (TIF) [file pcbi.1004522.s002.TIF]

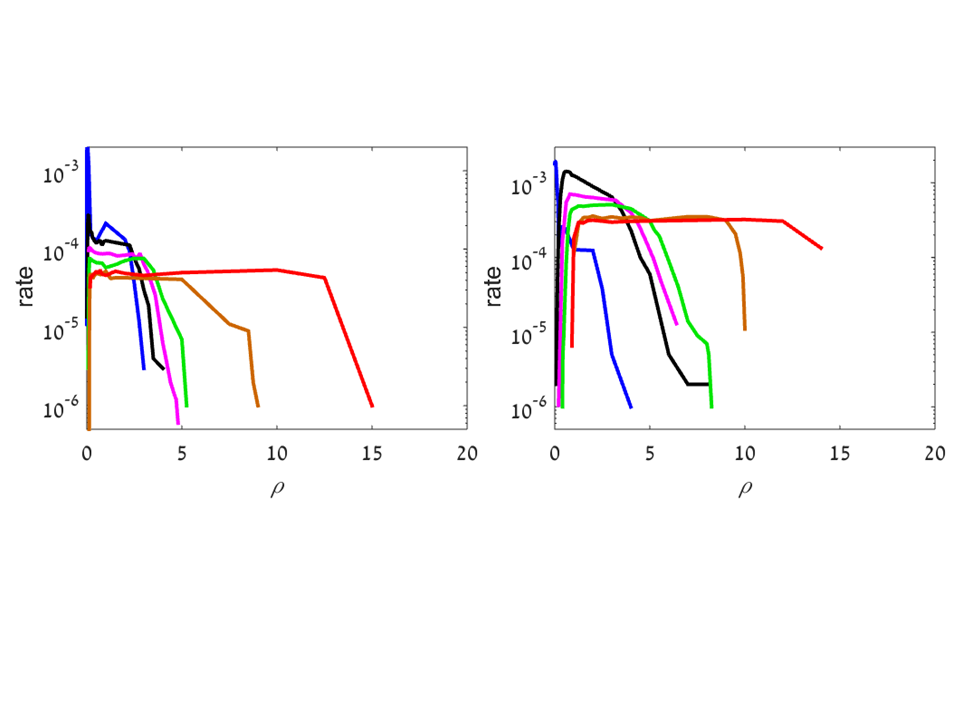

Supplement: S2 Fig — Blue: N = 20, Black: N = 50, Magenta: N = 100, Green: N = 200, Orange: N = 1000, Red: N = 2000. (TIF) [file pcbi.1004522.s003.TIF]

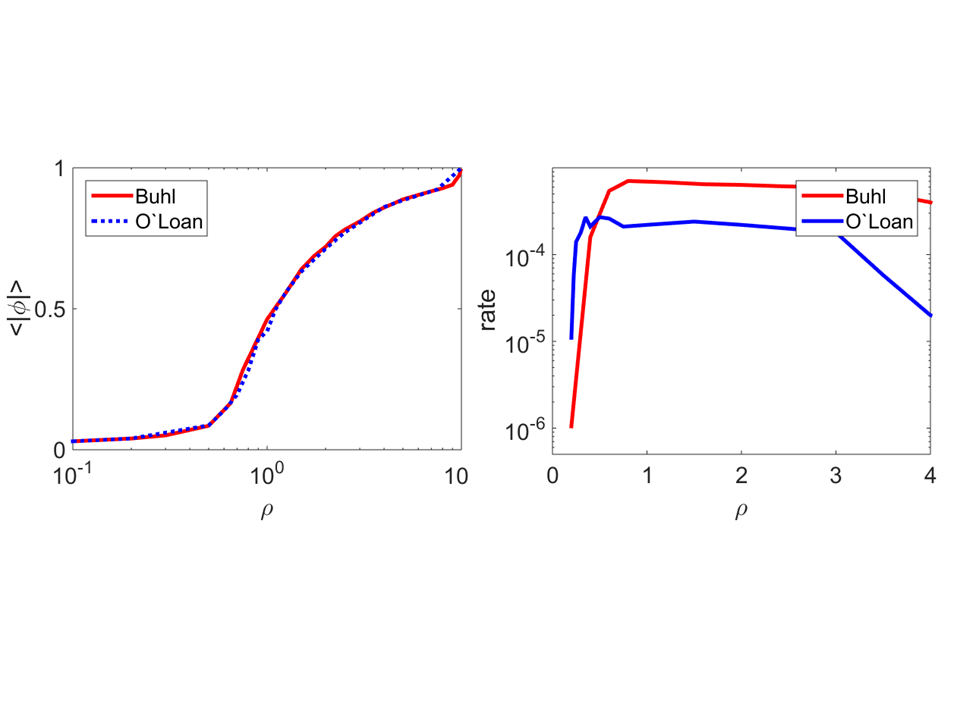

Supplement: S3 Fig — In the model of O`Loan and Evans [78] (blue lines), an animal makes a random choice whether to persist with its own speed or align with conspecifics. In the model of Buhl et al. [58] (red lines), individuals weigh their own speed separately with all others. Taking all other interactions and parameters to be the same, the two models result in practically the same average order parameter (left plot). Transition rates (right plot) with Buhl’s model are typically higher. N = 1000; all other parameters are the same as in Fig 2. (TIF) [file pcbi.1004522.s004.TIF]

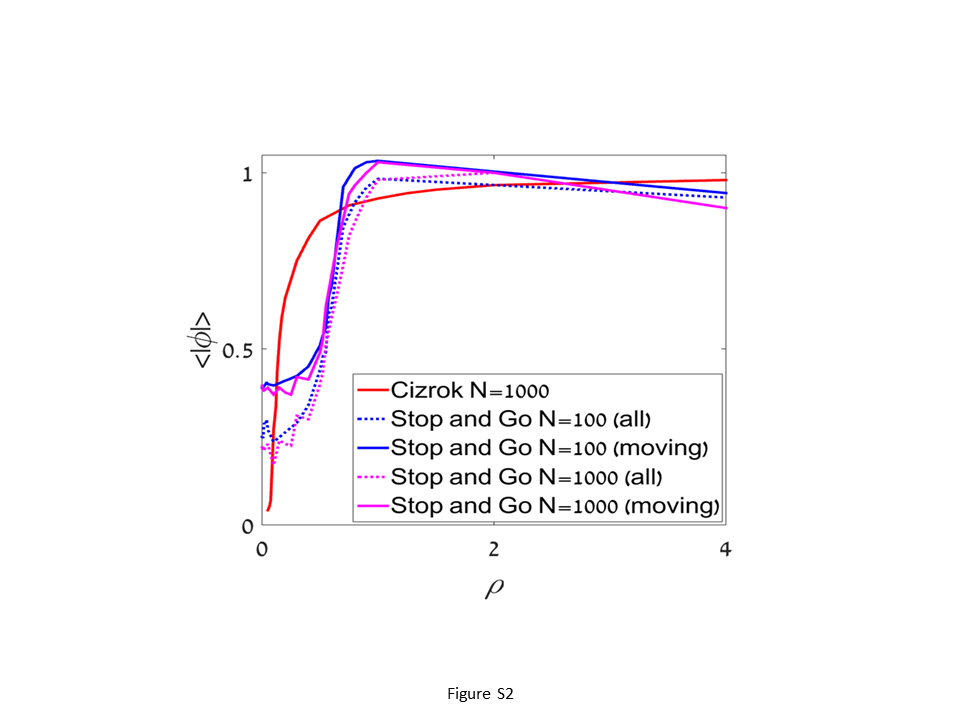

Supplement: S4 Fig — (all) The order parameter is calculated using all particles ϕ = 〈|(1 / N)Σi u i|〉. (moving) The order parameter is calculated using only moving particles ϕ = 〈(1 / N moving)Σi is moving u i〉. (TIF) [file pcbi.1004522.s005.TIF]

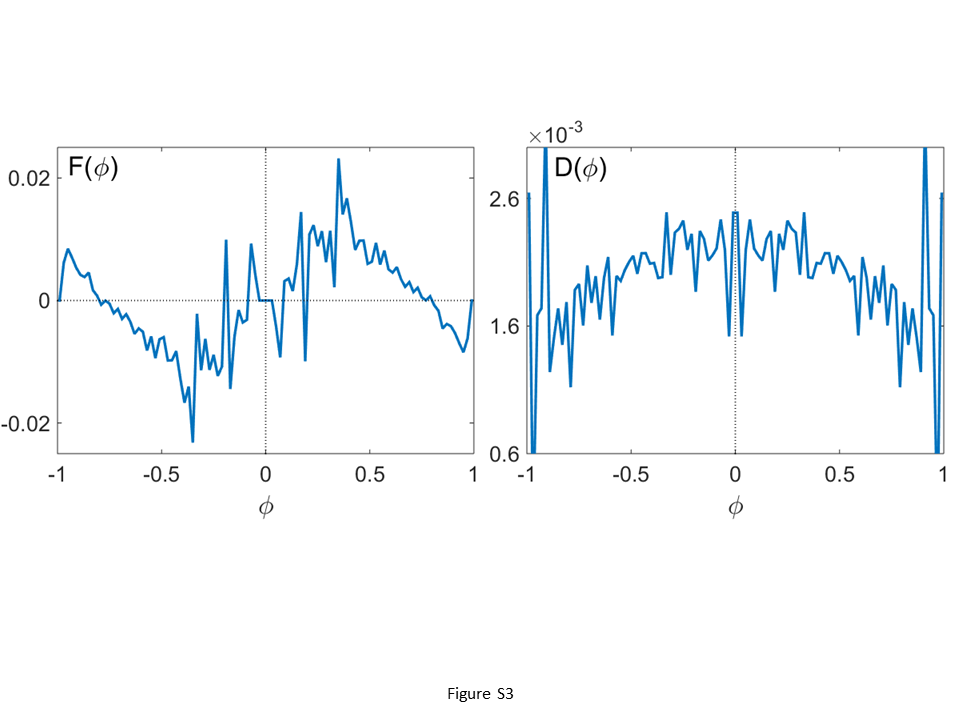

Supplement: S5 Fig — Adapted from [59]. The number of zeros of F(ϕ), corresponding to the metastable states of the system, is difficult to evaluate due to large statistical errors. (TIF) [file pcbi.1004522.s006.TIF]

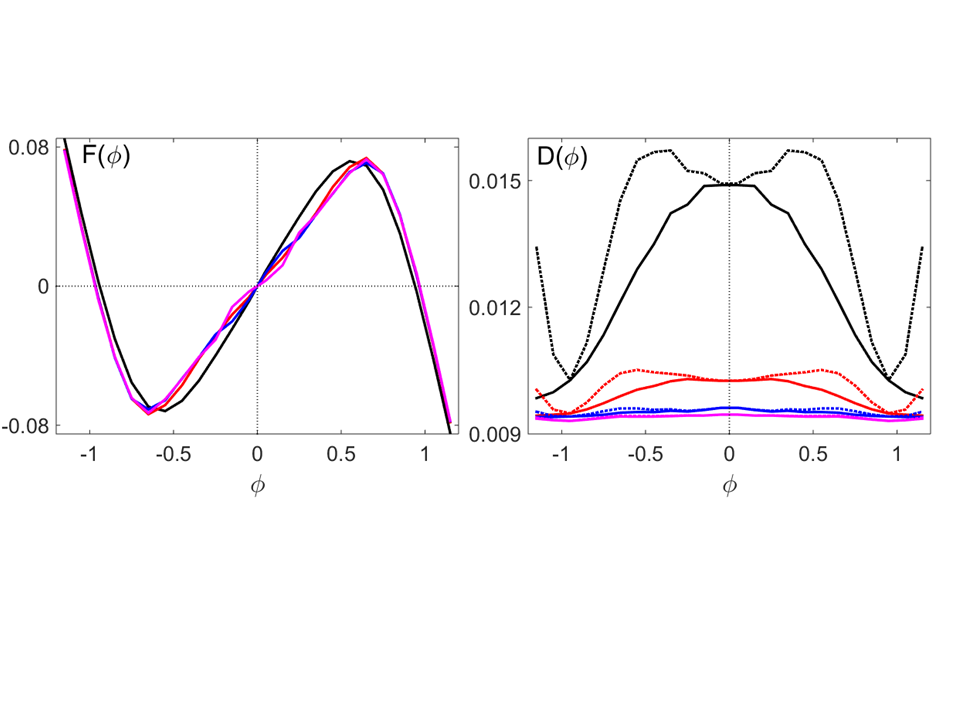

Supplement: S6 Fig — Solid line: using Eq 6. Dotted line: replacing the variance with the second moment. (TIF) [file pcbi.1004522.s007.TIF]

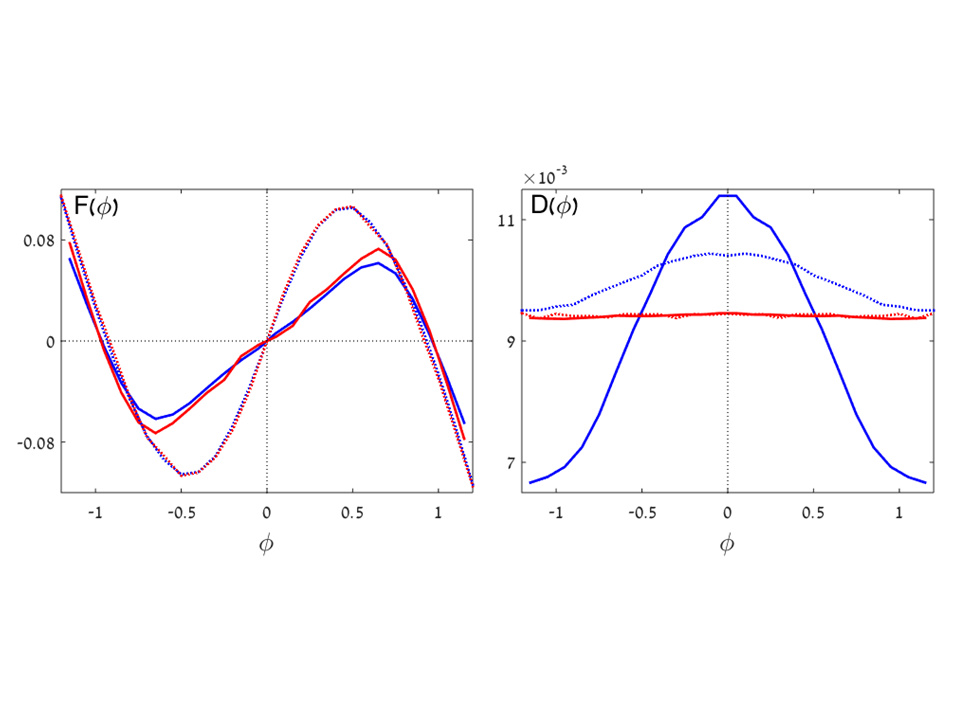

Supplement: S7 Fig — Red: Δt = 0.008, blue: Δt = 1. With very small Δt, the two sample methods yield practically the same effective diffusion but slightly different drift. However, with larger Δt, both the drift and diffusion functions are different. (TIF) [file pcbi.1004522.s008.TIF]
